# Plasma HMGB1 as a Potential Biomarker Reflecting the Clinical Outcome in Chronic Heart Failure Patients

**DOI:** 10.3390/jcm15031159

**Published:** 2026-02-02

**Authors:** Marcin Mazurek, Aneta Skwarek-Dziekanowska, Grzegorz Sobieszek, Teresa Małecka-Massalska, Tomasz Powrózek

**Affiliations:** 1 Department of Human Physiology of Chair of Preclinical Sciences, Medical University of Lublin, 20-059 Lublin, Polandtomaszpowrozek@gmail.com (T.P.); 2 Department of Cardiology, 1st Military Clinical Hospital with the Outpatient Clinic, 20-049 Lublin, Poland

**Keywords:** chronic heart failure, HMGB1, biomarker, inflammation, prognosis

## Abstract

**Background**: Chronic heart failure (CHF) is a progressive cardiovascular disease that predominantly affects elderly individuals and significantly impairs quality of life. High mobility group box 1 (HMGB1) has been proposed as a key mediator in the myocardial release of proinflammatory cytokines and the progression of CHF. The primary aim of this retrospective study was to evaluate the clinical significance of plasma HMGB1 levels in patients with CHF. The secondary objective was to determine the prognostic and predictive value of plasma HMGB1. **Methods**: Prior to the commencement of the study, blood samples were collected from 145 patients diagnosed with CHF. Plasma HMGB1 concentrations were measured at a single baseline time point using the enzyme-linked immunosorbent assay (ELISA). Statistical analyses were performed to assess correlations between HMGB1 levels and cardiac, laboratory, and nutritional parameters. **Results**: Elevated HMGB1 levels were significantly associated with worse clinical status, including increased pulmonary artery systolic pressure (PASP, *p* = 0.011), enlarged right ventricular outflow tract (RVOT, *p* = 0.006), advanced New York Heart Association (NYHA) functional class III or IV (*p* < 0.001), and the presence of dyspnea at rest (*p* < 0.001). HMGB1 levels effectively distinguished between NYHA classes I–III and IV (AUC = 0.780), as well as between cachectic and non-cachectic individuals (AUC = 0.840). Importantly, higher plasma HMGB1 concentrations were significantly associated with shorter overall survival (OS) in CHF patients (HR = 2.03; *p* < 0.001). **Conclusions**: Plasma HMGB1 levels may suggest that they reflect both cardiac and nutritional status in patients with CHF and could serve as a valuable biomarker for disease severity and prognosis. Notably, elevated HMGB1 is strongly associated with reduced overall survival, supporting its potential use in risk stratification and clinical management of CHF.

## 1. Introduction

Chronic heart failure (CHF) is recognized as one of the leading cardiovascular diseases worldwide, primarily impacting the elderly population [1,2]. In clinical practice, CHF is associated with elevated mortality rates, diminished quality of life, and a substantial financial burden on healthcare systems [3]. Within the first year following hospital admission, mortality among patients with CHF ranges from 17% to 45%, with rates exceeding 50% within five years of diagnosis [4]. Presently, clinical guidelines and laboratory tests, including the measurement of serum N-terminal pro-B-type natriuretic peptide (NT-proBNP), serve as the principal tools for diagnosing and monitoring CHF [5]. Emerging evidence indicated that president systemic inflammation plays a critical role in CHF pathophysiology, as reflected by circulating levels of C-reactive protein (CRP), tumor necrosis factor-alpha (TNF-α), and interleukin-6 (IL-6) [6]. Inflammation contributes to cardiomyocyte apoptosis and fibrosis, ultimately resulting in ventricular remodeling [7]. Various factors may exacerbate the inflammatory state observed in CHF patients, including comorbidities such as diabetes, atherosclerosis, and obesity, along with humoral responses and hemodynamic stress [8,9].

Cachexia is a common complication of CHF, driven by systemic inflammation. It is characterized by progressive weight loss, muscle wasting, and a systemic inflammatory response, affecting up to 40% of CHF patients, depending on diagnostic criteria [10,11]. Its progression is largely attributed to impaired myocardial perfusion, with inflammation playing a pivotal role. Alongside cachexia, malnutrition is also highly prevalent in this population, affecting approximately 75–90% of individuals with advanced disease or during episodes of exacerbation [12].

While existing clinical and biochemical markers are valuable for diagnosis, they typically alter only after structural or functional damage to cardiomyocytes has already occurred [13,14].

Nonetheless, a significant need persists in clinical practice for a robust biomarker that is easily measurable and possesses high accuracy in predicting clinical outcomes in individuals with CHF, extending beyond traditional risk factors. One such candidate is high mobility group box 1 (HMGB1), a protein implicated in the pathogenesis of numerous cardiovascular diseases, including coronary artery disease, heart failure, cardiac ischemia–reperfusion injury, and hypertension [15]. Biologically, HMGB1 is a non-histone chromatin-binding protein that is abundantly expressed in cardiomyocytes and cardiac tissue [16]. In recent years, HMGB1 has been determined to act as a damage-associated molecular pattern (DAMP) and extracellular proinflammatory mediator, released actively by stimulated immune cells, passively from necrotic or damaged cells. It activates neutrophils and dendritic cells, promoting the release of pro-inflammatory cytokines such as IL-6 and TNF-α by macrophages. Furthermore, HMGB1 interacts with the CXCL12/CXCR4 axis to activate the nuclear factor-κB (NF-κB) pathway, facilitating the chemotaxis and recruitment of inflammatory cells [17,18]. Systemic inflammation is now recognized as a key contributor to the onset, progression, and complications of CHF [19].

Despite its established role in cardiovascular disease, the clinical significance of HMGB1 in CHF, particularly regarding its association with systemic inflammation, nutritional status, and cachexia, remains unclear.

Importantly, inflammatory markers that are independent of echocardiographic findings, standard laboratory tests, or the New York Heart Association (NYHA) functional classification are regarded as significant prognostic and predictive indicators in patients with CHF [14]. In light of this context, the present study aimed to evaluate the relationship between plasma HMGB1 concentrations and the clinical characteristics of patients with CHF. A secondary objective was to assess the prognostic and predictive value of HMGB1 in this patient population.

## 2. Materials and Methods

### 2.1. Study Group

This retrospective study enrolled 145 patients (median age: 73 years; 86 males and 59 females) with newly diagnosed CHF (within the past 3 to 6 months). The study population included patients with CHF of ischemic origin, most commonly due to coronary artery disease (CAD). All participants were diagnosed and treated at the Clinic of Cardiology and Internal Medicine, Department of Cardiology, Military Hospital in Lublin, Poland, between 2015 and 2018. The diagnosis of CHF was in accordance with the 2012 European Society of Cardiology (ESC) guidelines, incorporating the 2016 update. The severity of heart failure was classified according to the New York Heart Association (NYHA) functional classification (classes I–IV). The study protocol was approved by the Bioethical Commission of the Medical University of Lublin, encompassing all procedures from sample collection to analysis of the obtained results (approval number: KE-0254/64/2017). This study was conducted in strict adherence to the principles of the Declaration of Helsinki. Written informed consent was obtained from all participants. To minimize potential bias, all clinical, laboratory, and nutritional assessments were performed using standardized protocols by trained personnel blinded to the study hypotheses. Each patient underwent a comprehensive routine clinical assessment, including a physical examination, echocardiography, laboratory testing, and nutritional evaluation. Echocardiographic measurements included left ventricular ejection fraction (LVEF), left atrial diameter (LAD), left ventricular end-diastolic diameter (LVEDd), left ventricular end-systolic diameter (LVESd), pulmonary artery systolic pressure (PASP), right ventricular outflow tract (RVOT) diameter, and tricuspid annular plane systolic excursion (TAPSE). Laboratory analyses included measurements of hemoglobin concentration, serum albumin, C-reactive protein (CRP), interleukin-6 (IL-6), tumor necrosis factor-alpha (TNF-α), and N-terminal pro–B-type natriuretic peptide (NT-proBNP), all performed during the routine examination of patients. All laboratory parameters were analyzed using standardized protocols with the Roche Cobas 6000 analyzer (Roche Diagnostics, Basel, Switzerland) and the Cormay Mythic 70 hematology analyzer (Cormay, Lublin, Poland). Inclusion criteria defined for this study were as follows: age > 18 years, a new diagnosis of CHF, and absence of clinical signs of tissue edema or fluid retention. Exclusion criteria comprised prior outpatient diagnoses of CHF, acute coronary syndrome, history of coronary artery bypass grafting, presence of an implanted cardioverter–defibrillator, and presence of thyroid disorders, end-stage kidney failure, active infection, autoimmune diseases, or previous or concomitant cancer.

Nutritional status was assessed using standardized clinical questionnaires—Subjective Global Assessment (SGA) and Nutritional Risk Score (NRS-2002)—and bioelectrical impedance analysis (BIA). The differentiation of patients into cachectic and non-cachectic was performed based on the Evans et al. criteria, extended by BIA (using ImpediMed bioimpedance SFB7 BioImp v1.55 device; Pinkenba, QLD, Australia). Body composition parameters were obtained from BIA provided data on fat mass (FM) and fat-free mass (FFM).

Fat-free mass index (FFMI) was calculated with the formula: FFMI [kg/m^2^] = FFM [kg]/(height [m])^2^. Cachexia in patients with CHF was diagnosed based on the presence of one primary criterion, unintentional body weight loss of ≥5% within the previous six months, along with at least three of the following five accessory criteria: (1) abnormal laboratory findings (e.g., elevated inflammatory markers, anemia, or hypoalbuminemia), (2) self-reported fatigue, (3) anorexia, (4) reduced muscle strength, and (5) low fat-free mass index (FFMI; <17.0 kg/m^2^ for males and <15.0 kg/m^2^ for females). According to these diagnostic criteria, cachexia was identified in 22.1% (95% CI = 15.60–29.70) of the study population. This study was designed based on the STROBE guidelines. A detailed clinical and demographic characteristics of the CHF cohort is presented in Table 1.

### 2.2. Analysis of HMGB1 Plasma Concentration

A 5 mL sample of peripheral blood was collected from each patient into EDTA-containing tubes. Then, the samples were centrifuged at 1000× *g* for 15 min to separate plasma, which was subsequently collected into cryotubes and stored at −80 °C until further laboratory analysis. The concentration of HMGB1 in plasma samples was measured in duplicate using an enzyme-linked immunosorbent assay (ELISA) with a commercially available kit (Human High Mobility Group Box 1 ELISA Kit, Cat. No. EEL047, Thermo Fisher Scientific, Waltham, MA, USA), following the manufacturer’s protocol. The intra-assay coefficients of variation (CV) were <10%, while the inter-assay CV were <10%. The assay had a detection range of 0–2000 pg/mL and a sensitivity of 18.75 pg/mL. Optical density was measured at 450 nm using a Multiskan FC microplate photometer (Thermo Scientific), and HMGB1 concentrations were calculated based on a standard curve generated using a four-parameter logistic (4-PL) regression model. An automated plate washer (Wellwash Versa, Thermo Scientific) was used for all washing steps. Plasma samples with HMGB1 concentrations exceeding the upper limit of detection were reanalyzed after appropriate dilution.

### 2.3. Statistical Analysis

Statistical analyses were performed using MedCalc software, version 15.8 (MedCalc Software, Oostende, Belgium). Differences in HMGB1 concentrations across clinical, demographic, cardiac, and laboratory variables were assessed using the Mann–Whitney U test. Univariate and multivariate logistic regression analyses were conducted to identify predictors of cachexia and NYHA functional class, with calculations of odds ratios (OR) and corresponding 95% confidence intervals (CI). Receiver operating characteristic (ROC) analysis was used to determine cut-off values and evaluate the diagnostic accuracy of HMGB1 in discriminating between patients based on cachexia status and disease severity (as defined by NYHA classification). Thresholds were derived and evaluated using the same dataset, which may represent a methodological limitation due to the potential for overly optimistic estimates of predictive performance. Correlations between HMGB1 levels and other studied variables were assessed using Spearman’s rank correlation coefficient (rho). The Kaplan–Meier estimator with the log-rank test and a Cox proportional hazard regression model were used for selection of variables affecting overall survival (OS) of the studied CHF patients. A *p*-value below 0.05 was considered statistically significant. For single statistical tests, unadjusted *p*-values were reported. In analyses involving multiple simultaneous tests, correction for multiple comparisons was applied, and statistical significance was assessed based on adjusted *p*-values.

## 3. Results

### 3.1. Relationships Between HMGB1 and Patients Outcomes

The classification of patients concerning each parameter was performed based on recommendations of American Society of Echocardiography (EF%, PASP, RVOT, LVEDd, LVESd, LAD, TAPSE) or median values (albumin, hemoglobin, CRP, IL-6, TNF-α, NT-proBNP, BMI, FM, FFM, FFMI). Patients classified to NYHA III or IV functional group demonstrated significantly elevated HMGB1 plasma levels (median: 6265.69 vs. 3814 pg/mL; *p* < 0.001) compared to those with NYHA I-II. Clinically, this suggests that HMGB1 may reflect the severity of CHF. Furthermore, higher HMGB1 levels were associated with an elevated PASP (median: 5737.36 vs. 3845.35 pg/mL; *p* = 0.011), increased RVOT (median: 6135.48 vs. 3797.54 pg/mL; *p* = 0.006) and the presence of dyspnea at rest (median: 6712.64 vs. 3831.02 pg/mL; *p* < 0.001). Our results suggest worse hemodynamic status and more advanced cardiac remodeling. HMGB1 concentration was significantly higher in CHF patients with anemia (median: 6712.64 vs. 3831.02 pg/mL; *p* = 0.0004), increased concentrations of CRP (median: 4340.43 vs. 3394.41 pg/mL; *p* = 0.005), IL-6 (median: 7568.23 vs. 5072.20 pg/mL; *p* = 0.018), TNF-α (median: 8141.32 vs. 3072.20 pg/mL; *p* = 0.023) and NT-proBNP (median: 5419.65 vs. 3853.54 pg/mL; *p* = 0.032). Thus, HMGB1 not only reflects cardiac dysfunction but also systemic inflammation and hematologic compromise, both of which contribute to poor prognosis in CHF.

Regarding nutritional status, plasma HMGB1 levels were significantly elevated in female patients with a lower FFMI (median: 7606.63 vs. 3676.03 pg/mL; *p* = 0.020). HMGB1 levels were also associated with SGA scores. Specifically, significantly higher HMGB1 concentrations were observed in patients with CHF classified as severely malnourished (SGA-C), compared to those categorized as mildly malnourished (SGA-B) or well-nourished (SGA-A) (median: 8412.03 vs. 4870.18 pg/mL; *p* = 0.028).

A similar trend was noted in CHF patients at higher nutritional risk according to the NRS-2002, where HMGB1 levels were significantly higher in those at risk (median: 8061.00 vs. 3845.35 pg/mL; *p* < 0.001). Furthermore, cachectic patients exhibited significantly elevated HMGB1 plasma concentrations compared to non-cachectic individuals (median: 8178.67 vs. 3859.74 pg/mL; *p* < 0.001) (Table 2). This indicates that HMGB1 may serve as a biomarker linking cardiac dysfunction with nutritional disorders, including cachexia, highlighting patients at higher probability for adverse outcomes.

### 3.2. Parameters Affecting Probability of Cachexia and Higher NYHA Classes

According to univariable analysis, patients with CHF classified as NYHA class III–IV had a significantly greater probability of cachexia, with an over 10-fold increased risk (OR = 10.19; *p* < 0.0001). A similar association was observed specifically for those in NYHA class IV (OR = 7.07; *p* < 0.001). Additionally, the like hood of cachexia was significantly higher in patients with hypoalbuminemia (OR = 3.78; *p* < 0.001), anemia (OR = 2.87; *p* = 0.010), and elevated CRP levels (OR = 10.53; *p* = 0.001). Increased plasma HMGB1 concentration also emerged as an independent predictor of cachexia (OR = 3.92; *p* = 0.002). In multivariable analysis, NYHA class III–IV (OR = 8.18; *p* = 0.005), low serum albumin levels (OR = 4.30; *p* < 0.001), elevated HMGB1 concentration (OR = 4.72; *p* = 0.029), and nutritional risk as assessed by NRS-2002 (OR = 8.85; *p* = 0.001) were identified as independent factors associated with the presence of cachexia in the study population. This underscores the importance of an integrated cardiological, laboratory, and nutritional assessment in the management of patients.

In univariable analysis, certain factors were identified as significant predictors of NYHA IV class. These included reduced EF% (OR = 4.42; *p* = 0.001), elevated PASP (OR = 2.90; *p* = 0.023), hypoalbuminemia (OR = 4.29; *p* < 0.001), increased CRP (OR = 8.75; *p* < 0.001), elevated NT-proBNP (OR = 5.39; *p* < 0.001), severe malnutrition as assessed by SGA (SGA-C) (OR = 4.86; *p* = 0.004), nutritional risk defined as NRS-2002 score ≥3 (OR = 3.88; *p* = 0.001), and elevated HMGB1 concentrations (OR = 4.27; *p* = 0.002). In multivariable analysis, elevated CRP (OR = 4.21; *p* = 0.004), increased NT-proBNP (OR = 4.26; *p* = 0.008), and higher plasma HMGB1 levels (OR = 2.99; *p* = 0.039) remained independently associated with NYHA IV class. Conversely, patients with increased RVOT measurements demonstrated a significantly lower likelihood, more than 4-fold of being classified as NYHA IV class (OR = 0.23; *p* = 0.005) (Table S1).

### 3.3. Diagnostic Accuracy of Plasma Levels of HMGB1 for CHF

**Figure 1:**
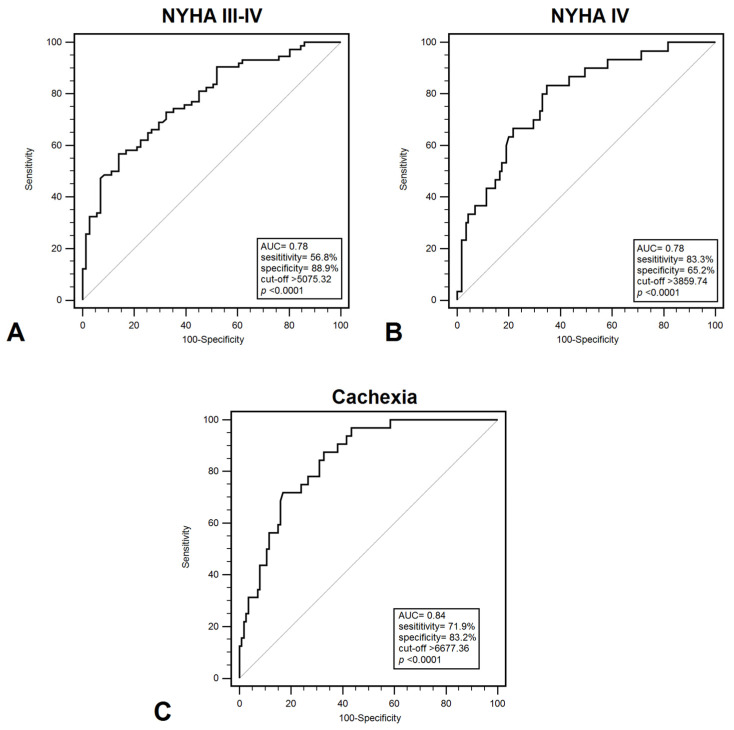
ROC curves demonstrating diagnostic accuracy of plasma HMGB1 values for discrimination between: (**A**) NYHA III –IV and NYHA I–II; (**B**) NYHA IV and NYHA I–III; (**C**) cachexia and non-cachexia. Abbreviations: AUC—area under curve; NYHA—New York Heart Association.

Plasma HMGB1 levels effectively differentiated between patients in NYHA class I–II and those in class III–IV, with a sensitivity of 56.8% and specificity of 85.9% (AUC = 0.78; *p* < 0.001) (Figure 1A). HMGB1 demonstrated a significant ability to discriminate between patients classified as NYHA class III and those in another NYHA classes, with a sensitivity of 55.7% and a specificity of 79.7% (AUC = 0.710; *p* = 0.0002). Furthermore, HMGB1 demonstrated discriminatory power in distinguishing between NYHA class I–III and class IV patients, with a sensitivity of 83.3% and specificity of 65.2% (AUC = 0.780; *p* < 0.001) (Figure 1B). Additionally, HMGB1 showed significant diagnostic value in identifying cachexia among CHF patients, yielding a sensitivity of 71.9% and specificity of 83.2% (AUC = 0.840; *p* < 0.001) (Figure 1C). These results indicate that HMGB1 may help identify patients with a higher probability of developing serious functional or nutritional disorders, which will allow for monitoring and implementation of earlier treatment. Diagnostic usefulness in distinguish patients with low FFMI (<19.77 kg/m^2^) demonstrated: 81.9% sensitivity and 67.7% specificity (AUC = 0.790; *p* < 0.001). On the other hand, the highest diagnostic accuracy were recorded for patients with low EF% (<40%) (AUC = 0.930; *p* < 0.001) and increased PASP (>36 mmHg) (AUC = 0.900; *p* < 0.001); however, EF% and PASP represent structural or functional measures rather than biomarkers (Table 3).

### 3.4. Correlation Between HMGB1 and Clinical Picture of CHF Patients

**Figure 2:**
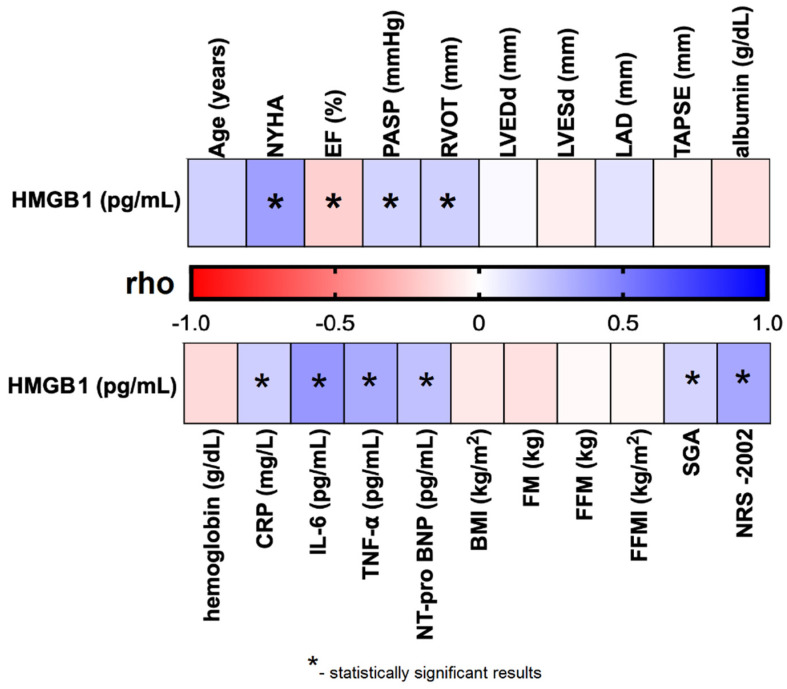
Correlation between plasma HMGB1 concentration and selected clinical parameters of the studied CHF patients. Abbreviations: BMI—body mass index; CRP—C reactive protein; EF%—ejection fraction; FFMI—fat-free mass index; FFM—fat-free mass; FM—fat mass; HMGB1—human high mobility group protein B1; IL–6—interleukin 6; LAD—left atrial diameter; LVEDd—left ventricular end-diastolic diameter; LVESd—left ventricular end-systolic diameter; NRS–2002—Nutritional Risk Score; NT-proBNP–N-terminal pro b-type natriuretic peptide; NYHA—New York Heart Association; PASP—pulmonary artery systolic pressure; rho—Spearman’s rank correlation coefficient; RVOT—right ventricular outflow tract; SGA—Subjective Global Assessment; TAPSE—tricuspid annular plane systolic excursion; TNF-α—tumor necrosis factor alpha.

We observed that plasma HMGB1 levels were significantly and negatively correlated with EF% (rho = –0.185; *p* = 0.026), and positively correlated with several cardiac parameters, including NYHA functional class (rho = 0.373; *p* < 0.001), PASP (rho = 0.170; *p* = 0.042), and RVOT (rho = 0.181; *p* = 0.030). Significant positive correlations were also found between HMGB1 levels and laboratory biomarkers: CRP (rho = 0.187; *p* = 0.026), IL-6 (rho = 0.405; *p* < 0.001), TNF-α (rho = 0.331; *p* = 0.004), and NT-proBNP (rho = 0.243; *p* = 0.003). Additionally, HMGB1 concentrations were significantly associated with nutritional assessments, including SGA classification (rho = 0.167; *p* = 0.044) and NRS-2002 score (rho = 0.344; *p* < 0.001) (Table S2; Figure 2).

### 3.5. Parameters Affecting Survival in Patients with CHF

**Figure 3:**
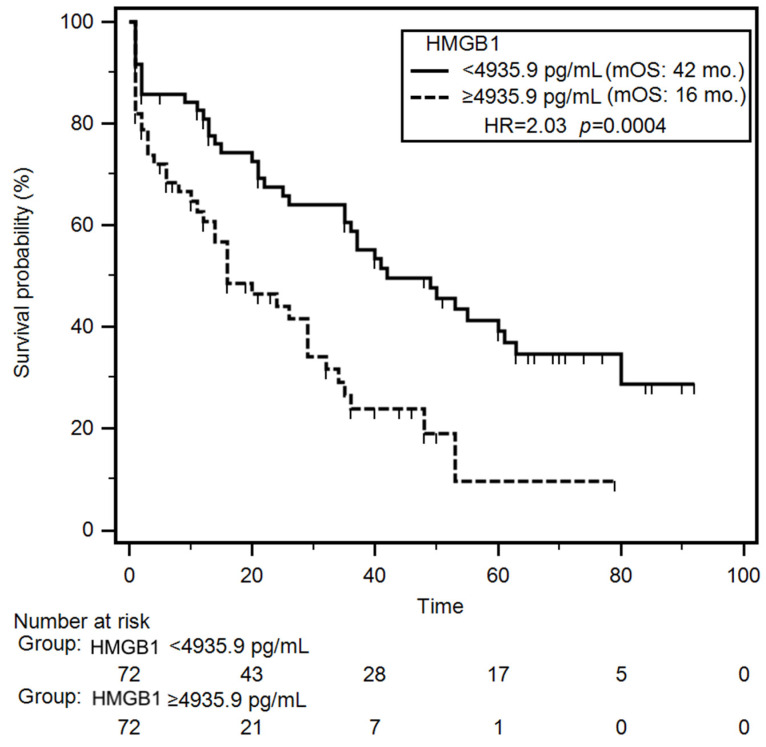
Differences in overall survival of CHF patients depending on median HMGB1 concentration. Abbreviations: HMGB1—human high mobility group protein B1; HR—hazard ratio; mOS—median overall survival.

Univariable survival analysis (Kaplan–Meier log-rank test) revealed that patients with NYHA class III–IV or class IV had a significantly increased risk of early mortality during the follow-up (HR = 1.78; *p* = 0.007 and HR = 2.27; *p* < 0.001, respectively). Additionally, patients with reduced TAPSE experienced significantly shorter median overall survival (OS) compared to those with preserved TAPSE (median OS: 21 months vs. 40 months; HR = 1.59; *p* = 0.031). Clinically, reduced TAPSE and advanced NYHA class identify patients at highest risk of mortality, emphasizing the need for intensive management. Laboratory parameters associated with significantly increased mortality risk included lower levels of serum albumin (HR = 1.96; *p* = 0.001), hemoglobin (HR = 1.83; *p* = 0.004), CRP (HR = 1.57; *p* = 0.036), TNF-α (HR = 1.82; *p* = 0.041), and NT-proBNP (HR = 1.61; *p* = 0.026). Notably, patients with elevated plasma HMGB1 concentrations had a significantly shorter median OS compared to those with lower HMGB1 levels (16 months vs. 42 months; HR = 2.03; *p* < 0.001) (Figure 3). Nutritional status also impacted survival outcomes. Severely malnourished patients (SGA-C) exhibited a higher risk of early death incidence (HR = 2.12; *p* < 0.001), as did those with increased nutritional risk (NRS-2002 score ≥3) (HR = 1.61; *p* = 0.028). In the group of patients demonstrating presence of cachexia median overall survival was almost 3-fold shorter (median: 11 months vs 37 months; HR = 2.14; *p* < 0.001) compared to non-cachectic patients. In multivariable Cox proportional hazards analysis, decreased TAPSE (HR = 1.57; *p* = 0.046), low serum albumin (HR = 1.83; *p* = 0.007), reduced hemoglobin levels (HR = 1.70; *p* = 0.020), elevated HMGB1 concentrations (HR = 1.96; *p* = 0.004) and coexisting cachexia (HR = 2.07; *p* = 0.007) emerged as independent predictors of poor survival. During the follow-up period, 82/145 (56.7%) patients died. The mortality rate was significantly higher (*p* = 0.043) in the group of patients with elevated concertation of plasma HMGB1 (43/65; 66.1%) than in group of other CHF patients (39/80; 48.7%). Results of the univariable and multivariable analyses of overall survival are summarized in Table 4 and Table S3.

## 4. Discussion

Despite substantial advancements in the treatment and management of cardiovascular diseases, the incidence of complications and the mortality rate associated with CHF have remained high in recent years [20]. Previous studies have explored various biomarkers to develop predictive and prognostic models for patients with cardiovascular conditions; however, these findings have yet to be integrated into routine clinical practice [21]. Consequently, the identification of novel biomarkers continues to be of significant clinical interest in the management of CHF. An ideal biomarker should fulfill several criteria: it must be detectable in blood samples from CHF patients, allow for rapid and reliable analysis, and exhibit sensitivity to cardiomyocyte damage and the associated inflammatory processes [6,22]. HMGB1, an extracellular protein, has been implicated in the pathophysiology of cardiovascular diseases and is considered a potential marker of both acute and chronic inflammation [23,24]. HMGB1 is a key mediator in both the initiation and amplification of inflammatory responses, classified within the family of DAMP molecules. Consequently, it has been extensively studied as an early indicator of inflammation in a range of acute and chronic inflammatory disorders. Increased levels of HMGB1 have been observed in serum, representing systemic inflammation, as well as in tissue-specific samples, reflecting local inflammatory activity in various infectious and inflammatory conditions [25]. In this study, we evaluated plasma concentrations of HMGB1 in patients with CHF and examined its correlations with selected cardiac (NYHA class, PASP, RVOT), laboratory (hemoglobin, CRP, IL-6, TNF-α, NT-proBNP), and nutritional parameters (FFMI, SGA, and NRS-2002). The observed relationship should be interpreted as observational and correlational rather than indicative of causality. Moreover, the study’s observational design may introduce potential confounding and reverse causality.

Recently, Volz et al. reported significantly higher HMGB1 concentrations in HF patients classified as NYHA functional class III-IV compared to those in class I–II (5.35 vs. 5.22 ng/mL; *p* < 0.001). Furthermore, among patients with ischemic cardiomyopathy, HMGB1 levels were insignificantly elevated compared to those with non-ischemic cardiomyopathy (4.70 vs. 4.37 ng/mL; *p* = 0.71) [26]. In another study involving 94 patients with CHF, serum HMGB1 levels were significantly higher in individuals with NYHA class IV compared to those in class II (*p* < 0.002). Additionally, markedly elevated HMGB1 concentrations were observed in HF patients relative to healthy controls (*p* = 0.025) [27]. Our findings align with these previous studies, as we also observed significantly higher concentrations of HMGB1 in patients with more advanced stages of CHF (NYHA class II–IV; *p* < 0.001). Furthermore, our analysis demonstrated that HMGB1 possesses predictive value for higher NYHA functional classification. Elevated plasma concentrations of HMGB1 were significantly associated with an increased likelihood of classification into NYHA class III–IV (OR = 8.18; *p* = 0.005) and specifically into class IV (OR = 4.47; *p* = 0.002). On the other hand, we observed association between RVOT and NYHA IV classification was counterintuitive (OR = 0.23; *p* = 0.005). This unexpected finding may be attributable to confounding factors, such as age or comorbidities, or to limitations inherent in RVOT measurement by echocardiography. Wahid et al. reported in ROC analysis that HMGB1 exhibited adequate accuracy for predicting the presence of HF with AUC of 0.861 (*p* < 0.001) [28]. We confirmed diagnostic accuracy in distinguishing between patients with various NYHA classes, demonstrating an AUC of 0.78 for NYHA I+II vs. III+IV and an AUC of 0.78 for NYHA I–III vs. IV. Moreover, we found that HMGB1 served as a predictor of low EF% (AUC = 0.93; *p* < 0.001) and increased PASP (AUC = 0.90; *p* < 0.001). In the study conducted by Wang et al., the authors demonstrated that patients with HF exhibited elevated levels of both HMGB1 and NT-proBNP, concluding that, unlike other parameters, high concentrations of HMGB1 and NT-proBNP may be associated with disease severity and clinical outcomes in HF [29]. Similarly, we observed that elevated HMGB1 levels were significantly associated with increased serum concentrations of NT-proBNP in patients with CHF (*p* = 0.032). The existing literature provides limited data on the relationship between HMGB1 and echocardiographic examinations. Notably, our results indicated that patients with higher HMGB1 levels also exhibited significantly elevated values of PASP (*p* = 0.011) and RVOT (*p* = 0.006). Recent studies have demonstrated that HMGB1 levels significantly correlate with established markers and predictors of HF, including NYHA functional classification (*p* < 0.001), NT-proBNP (*p* < 0.001), EF% (*p* < 0.001), creatinine (*p* = 0.005), and white blood cell count (WBC) (*p* < 0.001) [26]. Our findings corroborate these observations. We identified significant correlations between HMGB1 and NYHA functional class (*p* < 0.001), NT-proBNP (*p* = 0.003), EF% (*p* = 0.026), PASP (*p* = 0.042), and RVOT (*p* = 0.030), specifically in CHF patients.

The initiation of the inflammatory response is regarded as a crucial mechanism in the pathogenesis and progression of various cardiovascular diseases, including CHF. HMGB1 plays a central role in this process by stimulating both the inflammatory cascade and the activity of immune cells. This effect is primarily mediated through the activation of the NF-κB pathway, which subsequently leads to increased secretion of proinflammatory cytokines, such as TNF-α. We observed that elevated HMGB1 levels were significantly associated with increased plasma concentrations of TNF-α (*p* = 0.018) and IL-6 (*p* = 0.015) in patients with CHF. These findings support the role of HMGB1 in promoting the inflammatory response. Consistent with our results, a study by Yao et al. demonstrated a significant correlation between serum HMGB1 and CRP levels (*p* < 0.001), further confirming HMGB1’s involvement in inflammation [30]. In our study, HMGB1 levels were significantly correlated with CRP (*p* = 0.026), IL-6 (*p* < 0.001), and TNF-α (*p* = 0.004). Additionally, patients with elevated HMGB1 concentrations exhibited significantly higher CRP levels (*p* = 0.005). These findings underscore the contribution of HMGB1 to the inflammatory processes underlying CHF and suggest that this protein may play a pivotal role in the upregulation of other proinflammatory cytokines. Although statistical significance was achieved, the association was of moderate strength, limiting its immediate clinical relevance. Due to the observational nature of the study, we cannot conclude that HMGB1 mediates the effect through the inflammatory process. However, the presence of an inflammatory process may represent a confounding variable.

Patients with CHF frequently experience nutritional deficits, including cardiac cachexia [10,31]. In our study, 22.1% of participants were identified as cachectic. To date, only the study conducted by Ohmori et al. has reported elevated serum HMGB1 levels in cachectic patients, specifically in individuals with cancer, compared to non-cachectic controls (103.0 vs. 11.3 pg/mL; *p* < 0.001). In the context of cachexia, HMGB1 has been shown to participate in the regulation of myocyte autophagy via pathways involving receptor for advanced glycation end-products (RAGE) and Toll-like receptor 4 (TLR4) [32]. Supporting this mechanism, significantly elevated HMGB1 levels (1.8-fold increase; *p* < 0.001) were observed in the myocardium of CT26-inoculated BALB/c mice during cachexia progression [33]. In our study, elevated HMGB1 concentrations were also noted among CHF patients presenting with cachexia and poor nutritional status, as defined by classification C on the SGA and scores ≥3 on the NRS-2002. Importantly, we confirmed that higher HMGB1 levels were significantly associated with an increased probability of developing cachexia (OR = 3.92; *p* = 0.010). Moreover, HMGB1 demonstrated diagnostic utility in identifying cachexia among CHF patients, with an AUC of 0.84 (*p* < 0.001). It also exhibited diagnostic accuracy in distinguishing CHF patients with reduced FFMI, yielding an AUC of 0.79 (*p* < 0.001). Focusing on the prognostic value of HMGB1 in CHF, we found that elevated HMGB1 levels (≥4935.9 pg/mL) were associated with significantly shorter OS, with a median survival of 16 months compared to 42 months in patients with lower HMGB1 levels (<4935.9 pg/mL) (HR = 2.03; *p* = 0.004). This finding is consistent with the results reported by Kümmel et al., who indicated that patients with dilated cardiomyopathy and elevated HMGB1 levels (>0.53 ng/mL; n = 33) had a significantly lower survival probability compared to those with lower levels (≤0.53 ng/mL; n = 34) of this protein (univariate HR = 4.59; *p* = 0.032; multivariate HR = 1.92; *p* < 0.001) [34]. Similarly, another study in CHF patients identified elevated serum HMGB1 as a predictor of increased mortality risk (OR = 1.42) and reported significantly higher HMGB1 levels in non-survivors compared to survivors (median: 38.03 vs. 28.26 μg/L; *p* = 0.002) [27]. Furthermore, Volz et al. demonstrated that HMGB1, based on cut-off values of 3.4 and 6.1 ng/mL, was an independent negative prognostic marker for both mortality and heart transplantation (*p* = 0.001 and *p*<0.0001, respectively) in HF patients. In multivariable Cox regression models, HMGB1 concentrations above 3.4 ng/mL were associated with an increased risk of the combined endpoint of mortality and heart transplantation (HR = 2.48; 95% CI = 1.06–5.83; *p* = 0.037), while values exceeding 6.1 ng/mL were associated with an even higher risk (HR = 2.48; 95% CI = 1.31–4.71; *p* = 0.005) [26].

Measurement of plasma HMGB1 levels in patients with CHF may contribute to a more comprehensive understanding of the clinical presentation and prognosis of the disease. HMGB1 represents a noninvasive biomarker that enables straightforward concentration assessment without the need for specialized equipment, in contrast to genetic testing methods. Moreover, it may serve as a valuable adjunct to standard diagnostic approaches due to its potential widespread availability, rapid processing time, and cost-effectiveness in routine laboratory practice.

However, this study has several limitations. Further studies are required to assess and validate the specificity and clinical utility of the findings across diverse patient populations, including individuals with oncological conditions. The study was conducted at a single clinical center and included exclusively Polish patients, which may limit external validity due to reduced population heterogeneity and the potential influence of local environmental or healthcare-related factors, thereby increasing the risk of bias. Additionally, the retrospective design and relatively small sample size may further limit the generalizability of the results. The study also lacked external validation, did not include a healthy control group for comparison, and dichotomization may result in loss of information and a reduction in statistical power. Furthermore, the test should take into account additional parameters that would allow confirmation of the obtained results, such as troponin levels, interleukin-1 beta (IL-1β), galectin-3, or soluble suppression of tumorigenicity 2 (sST2). Therefore, further prospective studies with larger sample sizes are warranted to validate the clinical utility of HMGB1 as a biomarker in CHF.

## 5. Conclusions

Based on our findings, the analysis of plasma HMGB1 levels in patients with CHF appears to be a useful, robust, and sensitive marker for evaluating clinical status. We demonstrated that HMGB1 levels significantly correlate with NYHA functional class, inflammatory markers, nutritional status, and echocardiographic parameters. Moreover, HMGB1 not only reflects the degree of systemic inflammation but also shows potential as both a predictive and prognostic biomarker. Modulation of HMGB1 activity may enhance clinical outcomes and decrease the probability of cachexia in patients with CHF. The combined assessment of plasma HMGB1 and commonly used clinical biomarkers, such as NT-proBNP, may improve predictive and prognostic accuracy for clinical outcomes in CHF. Our results underscore the important role of HMGB1 in the pathophysiology of CHF, particularly in relation to inflammatory processes and nutritional disturbances, including cachexia. Future perspectives include investigating HMGB1-targeted therapeutic strategies to mitigate inflammation and prevent cachexia in CHF patients. Prospective studies are warranted to validate the clinical utility of HMGB1 as a routine biomarker for risk stratification and monitoring of CHF progression.

## Figures and Tables

**Table 1:** Baseline characteristics of the studied CHF patients.

Factor		*n* = 145 (100%)
Gender	Male	86 (59.3%)
	Female	59 (40.7%)
Age	Mean (range)	72 (27–95)
	<72 years	64 (44.1%)
	≥72 years	81 (55.9%)
Smoking status	Smoker	94 (64.8%)
	Non-smoker	51 (35.2%)
Diabetes	Yes	60 (41.4%)
	No	85 (58.6%)
Hyperlipidemia	Yes	58 (40%)
	No	87 (60%)
Kidney failure	Yes	50 (34.5%)
	No	95 (65.5%)
NYHA	I	27 (18.6%)
	II	44 (30.3%)
	III	44 (30.3%)
	IV	30 (20.7%)
Dyspnea	at rest	51 (35.2%)
	at exertion	131 (90.3%)
Cardiac arrhythmia	Yes	69 (47.6%)
	No	76 (52.4%)
Echocardiographic parameters		
EF%	Median (IQR)	42 (28.50–52)
	≤40%	73 (50.3%)
PASP (mmHg)	Median (IQR)	40 (32–48)
RVOT (mm)	Median (IQR)	34 (31–38)
LVEDd (mm)	Median (IQR)	55 (46.2–61.7)
LVESd (mm)	Median (IQR)	42 (38–50)
LAD (mm)	Median (IQR)	45 (40–50)
TAPSE (mm)	Median (IQR)	19 (15–20)
Laboratory testing		
Albumin (g/dL)	Median (IQR)	3.5 (3–3.80)
Hemoglobin (g/dL)	Median (IQR)	13.2 (11.4–14.35)
CRP (mg/L)	Median (IQR)	6 (2–22.15)
IL-6 (pg/mL)	Median (IQR)	7.16 (1.98–14.30)
TNF-α (pg/mL)	Median (IQR)	4.24 (3.10–5.69)
NT-proBNP (pg/mL)	Median (IQR)	2857 (1250–5285.5)
HMGB1 (pg/mL)	Median (IQR)	4935.9 (2469.65–8046.18)
Nutritional parameters		
BMI (kg/m^2^)	Median (IQR)	28.05 (25.1–32.4)
FM (kg)	Median (IQR)	25.72 (18.24–32.81)
FFM (kg)	Median (IQR)	54.11 (47.68–62.56)
FFMI (kg/m^2^)	Median (IQR)	19.77 (16.93–21.63)
SGA	A	66 (45.5%)
	B	63 (43.4%)
	C	16 (11%)
NRS—2002	≥3	46 (31.7%)
	<3	99 (68.3%)
Cachexia	Yes	32 (22.1%)
	No	113 (77.9%)

Abbreviations: BMI—body mass index; CRP—C reactive protein; EF%—ejection fraction; FFMI—fat-free mass index; FFM—fat-free mass; FM—fat mass; HMGB1—human high mobility group protein B1; IL-6—interleukin 6; IQR—interquartile range; LAD—left atrial diameter; LVEDd—left ventricular end-diastolic diameter; LVESd—left ventricular end-systolic diameter; NRS-2002—Nutritional Risk Score; NT-proBNP—N-terminal pro b-type natriuretic peptide; NYHA—New York Heart Association; PASP—pulmonary artery systolic pressure; RVOT—right ventricular outflow tract; SGA—Subjective Global Assessment; TAPSE—tricuspid annular plane systolic excursion; TNF-α—tumor necrosis factor alpha.

**Table 2:** Comparison of HMGB1 levels according to clinical-demographic characteristics, cardiac function, laboratory parameters, and nutritional status of CHF patients.

Parameter		HMGB1 Median (IQR)	*p*
Gender	Male	5017.07 (2469.85–7712)	0.859
	Female	4501 (2491.35–8531.79)	
Age (years)	<72	4761.89 (2307.56–6695)	0.119
	≥72	5295.84 (2644.43–8712.19)	
NYHA	I + II	3814 (1880.90–6645.78)	***<0.001***
	III + IV	6265.69 (3661–9208.91)	
	I–III	3845.35 (3358.76–5173.08)	***<0.001***
	IV	7797.25 (4935.90–5050.08)	
EF% (%)	<40	3816.69 (2469.65–7440.09)	0.059
	>40	5990.11 (2600.11–8704.87)	
PASP (mmHg)	>36	5737.36 (2940.81–8941.82)	***0.011***
	<36	3845.35 (2184.59–6807.71)	
RVOT (mm)	<33	3797.54 (2309.60–7610)	***0.006***
	>33	6135.48 (3687.76–8819.35)	
LVEDd (mm)	<57	4996.04 (2558.27–8044.11)	0.919
	>57	4935.90 (2397.23–7987.21)	
LVESd (mm)	<38	5171.15 (28320.15–9171.19)	0.262
	>38	4896.10 (2445.42–7610)	
LAD (mm)	<45	3676.03 (2363.17–5671.06)	0.621
	>45	3781.08 (2075.27–7875.68)	
TAPSE (mm)	<17	5246.98 (2754.80–8141.32)	0.368
	>17	4885.34 (3498.20–8668.17)	
Dyspnea at rest	Yes	6712.64 (3921–9069.61)	***<0.001***
	No	3831.02 (2249.63–6719)	
Dyspnea at exertion	Yes	4935.90 (2491.95–8043.98)	0.856
	No	4806.71 (2016.67–8052.40)	
Cardiac arrhythmia	Yes	4501 (2372.67–7931.62)	0.647
	No	5122.59 (2755.44–8048.26)	
Albumin (g/dL)	<3.2	5357.75 (2989.15–8668.17)	0.097
	>3.2	4531.49 (2372.67–7635.50)	
Hemoglobin (g/dL)	<12	6712.64 (3549.58–8921.44)	***0.048***
	>12	4761.89 (2364.16–7338.73)	
CRP (mg/L)	>5	4340.43 (2658.49–8986.41)	***0.005***
	<5	3394.41 (1870.18–5552.69)	
IL-6 (pg/mL)	>7.16	7568.23 (4935.90–9438.93)	***0.018***
	<7.16	5072.20 (3731.58–7729.66)	
TNF-α (pg/mL)	>4.24	7007.21(3751.93–11377.59)	***0.015***
	<4.24	4008.40 (2411.47–6686.18)	
NT-proBNP (pg/mL)	<2857	3853.54 (2219.60–6717.59)	***0.032***
	≥2857	5419.65 (3260.89–8660.89)	
BMI (kg/m^2^)	<24.9	4436.14 (3242.49–8043.58)	0.820
	>24.9	4959.80 (2469.25–8050.33)	
FM (kg)	<25.72	4320.79 (2622.33–8696.61)	0.216
	>25.72	4512.47 (2249.63–6712.64)	
FFM (kg)	<54.11	4512.47 (2377.51–8025.99)	0.765
	>54.11	3859.74 (2274.01–8009.93)	
FFMI (kg/m^2^) female	<17	7606.63 (3819.81–11369.68)	***0.020***
	>17	3676.03 (2169.78–5474.99)	
FFMI (kg/m^2^) male	<15	4653.61 (2571.53–7461.65)	0.693
	>15	4870.18 (2267.10–7839.88)	
SGA	A	4761.89 (2469.05–6712.64)	0.126
	B + C	5295.84 (2491.95–8819.35)	
SGA	A + B	4870.18 (2429.36–7752.89)	***0.028***
	C	8412.03 (3879.99–9167.60)	
NRS-2002	<3	3845.35 (2270.37–6670.34)	***<0.001***
	≥3	8061 (3939.98–12585.14)	
Cachexia	Yes	8178.67 (4653.62–10858.67)	***<0.001***
	No	3859.74 (2247.33–7140.87)	

Abbreviations: BMI—body mass index; CRP—C reactive protein; EF%—ejection fraction; FFMI—fat-free mass index; FFM—fat-free mass; FM—fat mass; HMGB1—human high mobility group protein B1; IL-6—interleukin 6; IQR—interquartile range; LAD—left atrial diameter; LVEDd—left ventricular end-diastolic diameter; LVESd—left ventricular end-systolic diameter; NRS-2002—Nutritional Risk Score; NT-proBNP–N-terminal pro b-type natriuretic peptide; NYHA—New York Heart Association; PASP—pulmonary artery systolic pressure; RVOT—right ventricular outflow tract; SGA—Subjective Global Assessment; TAPSE—tricuspid annular plane systolic excursion; TNF-α—tumor necrosis factor alpha.

**Table 3:** Diagnostic accuracy of the HMGPB-1 concentration for the discrimination of CHF patients depending on clinical and nutritional status occurrence.

Parameter	Sensitivity (%)	Specificity (%)	PPV (%)	NPV (%)	Cut-Off	AUC (95% CI) *p*
NYHA III	55.7	79.7	38.6	88.7	≤2249.63	0.710 (0.63–0.78) ***≤0.001***
NYHA IV	83.3	65.2	38.2	93.6	>3859.74	0.780 (0.71–0.85) ***<0.001***
NYHA III + IV	56.8	85.9	80.9	65.8	>5075.32	0.780 (0.70–0.84) ***<0.001***
EF% (%)	97.2	82.19	84.5	96.4	>3141.32	0.930 (0.88–0.97) ***<0.001***
PASP (mmHg)	82.7	84.4	87.7	78.2	>3511.49	0.900 (0.84–0.95) ***<0.001***
Dyspnea at rest	80.4	61.7	53.3	85.1	>3511.49	0.730 (0.65–0.81) ***<0.001***
FFMI (kg/m^2^)	81.9	67.7	74.9	76.4	>2949.86	0.790 (0.71–0.85) ***<0.001***
Cachexia	71.9	83.2	54.6	91.3	>6677.36	0.840 (0.78–0.90] ***<0.001***

Abbreviations: AUC—area under curve; CI—confidence intervals; EF%—ejection fraction; FFMI—fat-free mass index; NPV—negative predictive value; NYHA—New York Heart Association; PASP—pulmonary artery systolic pressure; PPV—positive predictive value.

**Table 4:** Significant parameters affecting OS of CHF patients selected by uni- and multivariate analysis.

Variable		Overall Survival				
		Univariable			Multivariable	
		mOS (Months)	HR (95% CI)	*p*	HR (95% CI)	*p*
NYHA	I + II III + IV	41 20	0.56 (0.36–0.87) 1.78 (1.15–2.75)	***0.007***	-	NS
NYHA	I + II + III IV	37 14	0.44 (0.24–0.81) 2.27 (1.23–4.19)	***<0.001***	-	NS
TAPSE (mm)	<17 >17	21 40	1.59 (0.99–2.54) 0.63 (0.40–1.01)	***0.031***	1.57 (1.01–2.45)	***0.046***
Albumin (g/dL)	<3.2 >3.2	14 40	1.96 (1.22–3.15) 0.51 (0.32–0.82)	***<0.001***	1.83 (1.18–2.85)	***0.007***
Hemoglobin (g/dL)	<12 >12	16 36	1.83 (1.15–2.92) 0.55 (0.34–0.87)	***0.004***	1.70 (1.09–2.64)	***0.020***
CRP (mg/L)	>5 <5	20 40	1.57 (1.02–2.43) 0.63 (0.41–0.98)	***0.036***	-	NS
TNF-α (pg/mL)	>4.24 <4.24	20 53	1.82 (0.99–3.32) 0.55 (0.0–1.00)	***0.041***	-	NS
NT-proBNP (pg/mL)	<2857 ≥2857	16 40	1.61 (1.03–2.49) 0.62 (0.40–0.96)	***0.026***	-	NS
HMGB1 (pg/mL)	<4935.9 ≥4935.9	42 16	0.49 (0.31–0.77) 2.03 (1.29–3.21)	***0.004***	1.96 (1.23–3.13)	***0.004***
SGA	C A or B	20 50	2.12 (1.38—3.28) 0.47 (0.31–0.73)	***<0.001***	-	NS
NRS-2002	<3 ≥3	37 16	0.62 (0.38–1.00) 1.61 (0.99–2.60)	***0.028***	-	NS
Cachexia	Yes No	11 37	2.14 (1.19–3.85) 0.47 (0.26–0.84)	***<0.001***	2.07 (1.21–3.52)	***0.007***

Abbreviations: CI—confidence intervals; CRP—C reactive protein; HMGB1—human high mobility group protein B1; HR—hazard ratio; NRS-2002—Nutritional Risk Score; NT-proBNP–N-terminal pro b-type natriuretic peptide; NS—non-significant; NYHA—New York Heart Association;; SGA—Subjective Global Assessment; TAPSE—tricuspid annular plane systolic excursion; TNF-α—tumor necrosis factor alpha.

## Data Availability

The original contributions presented in this study are included in this article. Further inquiries can be directed to the corresponding author.

## Supplementary Materials

The following supporting information can be downloaded at: https://www.mdpi.com/article/10.3390/jcm15031159/s1, Table S1. Cachexia and the occurrence of higher NYHA classification of CHF patients depending on selected demographic, cardiac, and laboratory variables; Table S2. Correlation between HMGB1 concertation and demographic, clinical and nutritional variables; Table S3. Influence of demographic, clinical, nutritional and laboratory variables on overall survival of CHF patients; Table S4. STROBE Statement—Checklist of items that should be included in reports of cohort studies.
