# Supplementary material for: Plasma HMGB1 as a Potential Biomarker Reflecting the Clinical Outcome in Chronic Heart Failure Patients
Source: J Clin Med. 2026 Feb 2;15(3):1159. doi: 10.3390/jcm15031159 (PMC12898523; doi:10.3390/jcm15031159)
Supplement: Supplementary file 1 [file jcm-15-01159-s001.zip › jcm-4109902-supplementary.doc]

**Table S1.** Cachexia and the occurrence of higher NYHA classification of CHF patients depending on selected demographic, cardiac, and laboratory variables

| **Variable** | **Cachexia** | | **NYHA III+IV** | | **NYHA IV** | |
| --- | --- | --- | --- | --- | --- | --- |
|  | **Univariable** | **Multivariable** | **Univariable** | **Multivariable** | **Univariable** | **Multivariable** |
|  | OR (95%CI)  *p* | OR (95%CI)  *p* | OR (95%CI)  *p* | OR (95%CI)  *p* | OR (95%CI)  *p* | OR (95%CI)  *p* |
| **NYHA**  (IV) | 7.07 (2.91-17.24)  ***<0.0001*** | 1.04 (0.22-4.78)  0.961 | NA | NA | NA | NA |
| **NYHA**  (III+IV) | 10.19 (3.35-31.02)  ***<0.0001*** | 8.18 (1.85-36.18)  ***0.005*** | NA | NA | NA | NA |
| **EF** (%) | 1.95 (0.87-4.37)  0.102 | 0.74 (0.18-2.94)  0.664 | 2.53 (1.29-4.94)  ***0.006*** | 1.44 (0.63-3.29)  0.387 | 4.42 (1.76-11.14)  ***0.001*** | 2.32 (0.78-6.84)  0.128 |
| **PASP** (mmHg) | 3.27 (1.31-8.16)  ***0.011*** | 1.45 (0.34-6.28)  0.616 | 4.78 (2.34-9.75)  ***<0.0001*** | 3.95 (1.72-9.11)  ***0.001*** | 2.90 (1.15-7.31)  ***0.023*** | 1.23 (0.40-3.77)  0.717 |
| **RVOT** (mm) | 0.53 (0.23-1.21)  0.131 | 1.61 (0.40-6.46)  0.499 | 0.78 (0.41-1.51)  0.465 | 1.22 (0.51-2.89)  0.650 | 0.16 (0.06-0.41)  ***0.0001*** | 0.23 (0.08-0.66)  ***0.005*** |
| **Albumin** (g/dL) | 3.78 (10.58-135.21)  ***<0.0001*** | 4.30 (8.82-212.71)  ***<0.0001*** | 2.85 (1.40-5.80)  ***0.003*** | 1.15 (0.46-2.86)  0.769 | 4.29 (1.85-9.99)  ***0.0007*** | 1.92 (0.67-5.51)  0.227 |
| **Hemoglobin** (g/dL) | 2.87 (1.28-6.42)  ***0.010*** | 1.35 (0.36-5.05)  0.654 | 1.89 (0.92-3.76)  0.081 | 0.93 (0.38-2.29)  0.877 | 0.59 (0.26-1.36)  0.217 | 1.05 (0.39-2.86)  0.923 |
| **CRP** (mg/L) | 10.53 (6.28-1764.79)  ***0.001*** | 81.72 (6.52-1023.35)  0.991 | 3.83 (1.92-7.63)  ***0.0001*** | 2.23 (0.96-5.21)  0.063 | 8.75 (2.87-2.62)  ***<0.0001*** | 4.21 (1.56-11.38)  ***0.004*** |
| **NT-proBNP** (pg/mL) | 4.84 (1.93-12.10)  ***0.0008*** | 0.80 (0.18-3.63)  0.777 | 5.63 (2.76-11.48)  ***<0.0001*** | 4.40 (1.96-9.88)  ***0.0003*** | 5.39 (2.05-14.18)  ***0.0006*** | 4.26 (1.45-12.54)  ***0.008*** |
| **HMGB1** (pg/mL) | 3.92 (1.62-9.47)  ***0.002*** | 4.72 (1.16-19.12)  ***0.029*** | 2.68 (1.37-5.24)  ***0.004*** | 2.02 (0.90-4.56)  0.088 | 4.27 (1.70-10.45)  ***0.002*** | 2.99 (1.05-8.51)  ***0.039*** |
| **SGA**  (B or C) | 19.59 (4.46-85.97)  ***0.0001*** | 1.14 (0.16-8.18)  0.900 | 2.12 (1.09-4.13)  ***0.026*** | 0.72 (0.30-1.72)  0.455 | 2.80 (1.15-6.80)  ***0.023*** | 1.30 (0.44-3.87)  0.636 |
| **SGA**  (C) | 8.11 (2.67-24.63)  ***0.0002*** | 2.48 (0.33-18.53)  0.375 | 17.80 (2.28-138.76)  ***0.006*** | 19.32 (2.15-173.87)  ***0.008*** | 4.86 (1.65-14.35)  ***0.004*** | 2.51 (0.62-10.27)  0.200 |
| **NRS-2002** | 15.65 (5.97-40.98)  ***<0.0001*** | 8.58 (2.33-31.58)  ***0.001*** | 3.59 (1.68-7.65)  ***0.0009*** | 0.72 (0.25-2.12)  0.548 | 3.88 (1.68-8.95)  ***0.001*** | 1.25 (0.42-3.72)  0.696 |

Abbreviations: CI – confidence interval; CRP – C reactive protein; EF%—ejection fraction; HMGB1 – human high mobility group protein B1; NA – not applicable; NRS-2002—Nutritional Risk Score; NT-proBNP– N-terminal pro b-type natriuretic peptide; NYHA—New York Heart Association; OR – odds ratio; PASP – pulmonary artery systolic pressure; RVOT – right ventricular outflow tract; SGA—Subjective Global Assessment

**Table S2.** Correlation between HMGB1 concertation and demographic, clinical and nutritional variables

| **Factor** | **HMGB1** | |
| --- | --- | --- |
|  | **rho**  **[95%CI]** | ***p*** |
| **Age** (years) | 0.178  [0.01-0.33] | 0.326 |
| **NYHA** | 0.37  [0.22-0.51] | ***<0.0001*** |
| **EF** (%) | -0.185  [-0.33-0.31] | ***0.026*** |
| **PASP** (mmHg) | 0.170  [0.006-0.32] | ***0.042*** |
| **RVOT** (mm) | 0.181  [0.02-0.33] | ***0.030*** |
| **LVEDd** (mm) | 0.023  [-0.14-0.19] | 0.781 |
| **LVESd** (mm) | -0.066  [-0.29-0.09] | 0.431 |
| **LAD** (mm) | 0.109  [-0.06-0.27] | 0196 |
| **TAPSE** (mm) | -0.045  [-0.21-0.12] | 0.588 |
| **Albumin** (g/dL) | -0.118  [-0.28-0.05] | 0.156 |
| **Hemoglobin** (g/dL) | -0.148  [-0.30-0.02] | 0.075 |
| **CRP** (mg/L) | 0.187  [0.03-0.34] | ***0.026*** |
| **IL-6** (pg/mL) | 0.405  [0.18-0.59] | ***0.0006*** |
| **TNF-α** (pg/mL) | 0.331  [0.11-0.52] | ***0.004*** |
| **NT-proBNP** (pg/mL) | 0.243  [0.08-0.39] | ***0.003*** |
| **BMI** (kg/m^2^) | -0.009  [-0.25-0.07] | 0.264 |
| **FM** (kg) | -0.119  [-0.29-0.006] | 0.198 |
| **FFM** (kg) | -0.024  [-0.20-0.16] | 0.791 |
| **FFMI** (kg/m^2^) | -0.037  [-0.22-0.15] | 0.690 |
| **SGA** | 0.167  [0.004-0.32] | ***0.044*** |
| **NRS-2002** | 0.344  [0.19-0.48] | ***<0.0001*** |

Abbreviations: CI – confidence interval; BMI – body mass index; CRP – C reactive protein; EF%—ejection fraction; FFMI – fat-free mass index; FFM—fat-free mass; FM—fat mass; HMGB1 – human high mobility group protein B1; IL-6 – interleukin 6; LAD – left atrial diameter; LVEDd – left ventricular end-diastolic diameter; LVESd – left ventricular end-systolic diameter; NRS-2002—Nutritional Risk Score; NT-proBNP– N-terminal pro b-type natriuretic peptide; NYHA—New York Heart Association; PASP – pulmonary artery systolic pressure; RVOT – right ventricular outflow tract; SGA—Subjective Global Assessment; TAPSE—tricuspid annular plane systolic excursion; TNF-α – tumor necrosis factor alpha

**Table S3.** Influence of demographic, clinical, nutritional and laboratory variables on overall survival of CHF patients

| **Variable** | | **Overall survival** | | | | |
| --- | --- | --- | --- | --- | --- | --- |
|  |  | **Univariable** | | | **Multivariable** | |
|  |  | mOS (months) | HR (95% CI) | *p* | HR (95% CI) | *p* |
| **Gender** | Men  Women | 26  36 | 1.06 (0.68-1.65)  0.94 (0.61-1.46) | 0.780 | 0.94 (0.57-1.51) | 0.768 |
| **Age** (years) | <72  ≥72 | 29  48 | 1.82 (1.17-2.82)  0.55 (0.36-0.85) | 0.071 | 2.08 (1.19-3.63) | 0.080 |
| **Smoking status** | Smoker  Non- smoker | 26  32 | 1.05 (0.67-1.66)  0.95 (0.60-1.49) | 0.820 | 1.17 (0.73-1.88) | 0.523 |
| **Diabetes** | Yes  No | 34  32 | 1.08 (0.70-1.67)  0.93 (0.60-1.43) | 0.716 | 1.05 (0.67-1.66) | 0.817 |
| **Hyperlipidemia** | Yes  No | 42  24 | 0.67 (0.43-1.03)  1.49 (0.97-2.31) | 0.072 | 0.77 (0.48-1.23) | 0.284 |
| **Kidney failure** | Yes  No | 19  43 | 1.06 (0.67-1.65)  0.95 (0.61-1.49) | 0.823 | 1.07 (0.68-1.67) | 0.779 |
| **NYHA** | I+II  III+IV | 41  20 | 0.56 (0.36-0.87)  1.78 (1.15-2.75) | ***0.007*** | 1.31 (0.81-2.10) | 0.270 |
| **NYHA** | I+II+III  IV | 37  14 | 0.44 (0.24-0.81)  2.27 (1.23-4.19) | ***<0.001*** | 1.45 (0.83-2.54) | 0.190 |
| **EF** (%) | ≤40  >40 | 24  49 | 1.41 (0.91-2.18)  0.71 (0.56-1.09) | 0.105 | 1.23 (0.75-2.02) | 0.418 |
| **RVOT** (mm) | <33  >33 | 36  26 | 0.93 (0.61-1.44)  1.07 (0.69-1.65) | 0.752 | 1.21 (0.76-1.94) | 0.420 |
| **TAPSE** (mm) | <17  >17 | 21  40 | 1.59 (0.99-2.54)  0.63 (0.40-1.01) | ***0.031*** | 1.57 (1.01-2.45) | ***0.046*** |
| **PASP** (mmHg) | <36  >36 | 25  37 | 1.11 (0.72-1.73)  0.90 (0.58-1.39) | 0.623 | 0.65 (0.40-1.07) | 0.096 |
| **LVEDd** (mm) | <57  >57 | 29  32 | 1.44 (0.93-2.23)  0.69 (0.45-1.07) | 0.103 | 0.69 (0.43-1.09) | 0.113 |
| **LVESd** (mm) | <38  >38 | 29  32 | 1.27 (0.83-1.96)  0.79 (0.51-1.22) | 0.269 | 0.79 (0.51-1.22) | 0.281 |
| **LAD** (mm) | <45  >45 | 34  32 | 0.82 (0.53-1.27)  1.22 (0.79-1.89) | 0.351 | 0.89 (0.53-1.26) | 0.363 |
| **Albumin** (g/dL) | <3.2  >3.2 | 14  40 | 1.96 (1.22-3.15)  0.51 (0.32-0.82) | ***<0.001*** | 1.83 (1.18-2.85) | ***0.007*** |
| **Hemoglobin** (g/dL) | <12  >12 | 16  36 | 1.83 (1.15-2.92)  0.55 (0.34-0.87) | ***0.004*** | 1.70 (1.09-2.64) | ***0.020*** |
| **CRP** (mg/L) | >5  <5 | 20  40 | 1.57 (1.02-2.43)  0.63 (0.41-0.98) | ***0.036*** | 1.39 (0.88-2.22) | 0.163 |
| **IL-6** (pg/mL) | >7.16  <7.16 | 20  36 | 1.51 (0.80-2.84)  0.66 (0.35-1.25) | 0.185 | 0.87 (0.39-1.96) | 0.741 |
| **TNF-α** (pg/mL) | >4.24  <4.24 | 20  53 | 1.82 (0.99-3.32)  0.55 (0.0-1.00) | ***0.041*** | 0.87 (0.39-1.93) | 0.727 |
| **NT-proBNP** (pg/mL) | <2857  ≥2857 | 16  40 | 1.61 (1.03-2.49)  0.62 (0.40-0.96) | ***0.026*** | 1.20 (0.75-1.92) | 0.447 |
| **HMGB1** (pg/mL) | <4935.9  ≥4935.9 | 42  16 | 0.49 (0.31-0.77)  2.03 (1.29-3.21) | ***0.004*** | 1.96 (1.23-3.13) | ***0.004*** |
| **SGA** | B or C  A | 29  32 | 1.24 (0.62-2.47)  0.81 (0.41-1.61) | 0.495 | 0.67 (0.33-1.33) | 0.252 |
| **SGA** | C  A or B | 20  50 | 2.12 (1.38—3.28)  0.47 (0.31-0.73) | ***<0.001*** | 1.29 (0.72-2.33) | 0.397 |
| **NRS-2002** | <3  ≥3 | 37  16 | 0.62 (0.38-1.00)  1.61 (0.99-2.60) | ***0.028*** | 0.95 (0.57-1.58) | 0.837 |
| **Cachexia** | Yes  No | 11  37 | 2.14 (1.19-3.85)  0.47 (0.26-0.84) | ***<0.001*** | 2.07 (1.21-3.52) | ***0.007*** |

Abbreviations: CI -confidence intervals; CRP – C reactive protein; EF%—ejection fraction; HMGB1 – human high mobility group protein B1; HR – hazard ratio; IL-6 – interleukin 6; LAD – left atrial diameter; LVEDd – left ventricular end-diastolic diameter; LVESd – left ventricular end-systolic diameter; NRS-2002—Nutritional Risk Score; NT-proBNP– N-terminal pro b-type natriuretic peptide; NYHA—New York Heart Association; PASP – pulmonary artery systolic pressure; RVOT – right ventricular outflow tract; OS – overall survival; SGA—Subjective Global Assessment; TAPSE—tricuspid annular plane systolic excursion; TNF-α – tumor necrosis factor alpha

**Table S4.** STROBE Statement—Checklist of items that should be included in reports of ***cohort studies***

|  | Item No | Recommendation | Page No. |
| --- | --- | --- | --- |
| **Title and abstract** | 1 | (*a*) Indicate the study’s design with a commonly used term in the title or the abstract | 1 |
|  |  | (*b*) Provide in the abstract an informative and balanced summary of what was done and what was found | 1 |
| Introduction | | |  |
| Background/rationale | 2 | Explain the scientific background and rationale for the investigation being reported | 1-2 |
| Objectives | 3 | State specific objectives, including any prespecified hypotheses | 2 |
| Methods | | |  |
| Study design | 4 | Present key elements of study design early in the paper | 3 |
| Setting | 5 | Describe the setting, locations, and relevant dates, including periods of recruitment, exposure, follow-up, and data collection | 3 |
| Participants | 6 | (*a*) Give the eligibility criteria, and the sources and methods of selection of participants. Describe methods of follow-up | 3-4 |
|  |  | (*b*) For matched studies, give matching criteria and number of exposed and unexposed | *n/a* |
| Variables | 7 | Clearly define all outcomes, exposures, predictors, potential confounders, and effect modifiers. Give diagnostic criteria, if applicable | 6 |
| Data sources/ measurement | 8* | For each variable of interest, give sources of data and details of methods of assessment (measurement). Describe comparability of assessment methods if there is more than one group | 4-6 |
| Bias | 9 | Describe any efforts to address potential sources of bias | 16 |
| Study size | 10 | Explain how the study size was arrived at | 6 |
| Quantitative variables | 11 | Explain how quantitative variables were handled in the analyses. If applicable, describe which groupings were chosen and why | *n/a* |
| Statistical methods | 12 | (*a*) Describe all statistical methods, including those used to control for confounding | 5 |
|  |  | (*b*) Describe any methods used to examine subgroups and interactions | 5 |
|  |  | (*c*) Explain how missing data were addressed | *n/a* |
|  |  | (*d*) If applicable, explain how loss to follow-up was addressed | *n/a* |
|  |  | (*e*) Describe any sensitivity analyses | *n/a* |
| Results | | |  |
| Participants | 13* | (a) Report numbers of individuals at each stage of study—eg numbers potentially eligible, examined for eligibility, confirmed eligible, included in the study, completing follow-up, and analysed | 6 |
|  |  | (b) Give reasons for non-participation at each stage | *n/a* |
|  |  | (c) Consider use of a flow diagram | *n/a* |
| Descriptive data | 14* | (a) Give characteristics of study participants (eg demographic, clinical, social) and information on exposures and potential confounders | 6-8 |
|  |  | (b) Indicate number of participants with missing data for each variable of interest | Tables |
|  |  | (c) Summarise follow-up time (eg, average and total amount) | *n/a* |
| Outcome data | 15* | Report numbers of outcome events or summary measures over time | Tables |
| Main results | 16 | (*a*) Give unadjusted estimates and, if applicable, confounder-adjusted estimates and their precision (eg, 95% confidence interval). Make clear which confounders were adjusted for and why they were included | Tables |
|  |  | (*b*) Report category boundaries when continuous variables were categorized | 3-5 |
|  |  | (*c*) If relevant, consider translating estimates of relative risk into absolute risk for a meaningful time period | *n/a* |
| Other analyses | 17 | Report other analyses done—eg analyses of subgroups and interactions, and sensitivity analyses | 3-5 |
| Discussion | | |  |
| Key results | 18 | Summarise key results with reference to study objectives | 13 |
| Limitations | 19 | Discuss limitations of the study, taking into account sources of potential bias or imprecision. Discuss both direction and magnitude of any potential bias | 15 |
| Interpretation | 20 | Give a cautious overall interpretation of results considering objectives, limitations, multiplicity of analyses, results from similar studies, and other relevant evidence | 12-15 |
| Generalisability | 21 | Discuss the generalisability (external validity) of the study results | 13 |
| Other information | | |  |
| Funding | 22 | Give the source of funding and the role of the funders for the present study and, if applicable, for the original study on which the present article is based | 15 |

*Give information separately for exposed and unexposed groups.
